# Investigation of Relationships between Intakes of Human Milk Total Lipids and Metabolic Hormones and Infant Sex and Body Composition

**DOI:** 10.3390/nu16162739

**Published:** 2024-08-16

**Authors:** Majed A. Suwaydi, Ching Tat Lai, Ashleigh H. Warden, Sharon L. Perrella, Jacki L. McEachran, Mary E. Wlodek, Donna T. Geddes, Zoya Gridneva

**Affiliations:** 1 School of Molecular Sciences, The University of Western Australia, Crawley, WA 6009, Australia; majed.suwaydi@research.uwa.edu.au (M.A.S.); ching-tat.lai@uwa.edu.au (C.T.L.); ashleigh.warden@uwa.edu.au (A.H.W.); sharon.perrella@uwa.edu.au (S.L.P.); jacki.mceachran@uwa.edu.au (J.L.M.); mary.wlodek@uwa.edu.au (M.E.W.); donna.geddes@uwa.edu.au (D.T.G.); 2 School of Applied Medical Sciences, Jazan University, Jazan 45142, Saudi Arabia; 3 ABREAST Network, Perth, WA 6000, Australia; 4 UWA Centre for Human Lactation Research and Translation, Crawley, WA 6009, Australia; 5 Department of Obstetrics, Gynaecology and Newborn Health, The University of Melbourne, Parkville, VIC 3010, Australia

**Keywords:** human milk, lactation, leptin, adiponectin, insulin, total lipids, intake, body composition, infant, adiposity

## Abstract

Human milk (HM) composition, including metabolic hormones and lipids, is influenced by various factors, including lactation stage and, potentially, infant sex, which may affect infant body composition (BC) development. We aimed to: (a) characterize the longitudinal concentration and intake profiles of HM leptin, adiponectin, insulin, and total lipids; (b) determine if their concentrations and intakes differ by infant sex; and (c) explore the intakes relationships with the development of infant BC. Milk samples (*n* = 501) were collected from 82 mother–infant dyads during the first 6 months postpartum. Infant 24 h HM intake was measured, and the average cumulative HM component intakes were calculated. The statistical analysis used linear mixed modeling. Intakes of HM leptin, adiponectin, insulin, and total lipids increased to 1 month postpartum and then remained stable. HM intake and total lipids intake but not hormone intakes were positively associated with infant BC (fat-free mass, fat-free mass index, fat mass, fat mass index, percentage fat mass, and fat mass to fat-free mass ratio). HM component concentrations and intakes did not differ by sex. These findings advance our understanding of the temporal nature of HM components, emphasizing the role of infant 24 h HM and total lipids intake in development of infant lean and adipose tissue.

## 1. Introduction

Human milk (HM) provides infants with an optimal albeit dynamic nutritional and bioactive profile that is suitable for their growth and development. The concept of HM as a biological system has become a focus of extensive research in the past decade [1,2,3,4]. HM composition, including metabolic hormones and fat, is influenced by lactation stage, circadian rhythm, and within-feed factors. HM leptin, adiponectin, insulin, and total lipids concentrations have been reported to vary throughout lactation [5,6,7,8]. It has been surmised that the infants’ nutritional and developmental demands might be the regulators of milk component variation over lactation [9], along with genetic, nutritional, and environmental factors [10]. In this context, variations in HM composition may play a role in infant growth, body composition (BC) development, metabolism, and long-term health outcomes.

Different studies have aimed to characterize the longitudinal variation in HM components, such as total lipids and leptin. These studies provide an opportunity to understand the impact of HM components on the developing infant; however, rarely are components measured in whole milk, nor are intakes of the components routinely measured. Recently, it has been shown that the concentration of total HM lipids did not change over the first 6 months of lactation; however, significant changes from colostrum to mature milk in many bioactive lipids, such as ether lipids and gangliosides that possess various biological functions, have been reported [11]. HM leptin is known to decrease from colostrum to 6 weeks postpartum [12], and a cross-sectional study has indicated a decrease in concentration up to 6 months [13]. Further, studies have reported conflicting results regarding changes in HM adiponectin concentration over the lactation duration [14,15]. It is, therefore, essential to study the longitudinal variation in HM component concentrations and intakes over the exclusive lactation period along with maternal determinants to understand the mechanisms by which HM intake supports optimal infant growth and development.

Maternal anthropometric and BC measurements are important determinants of HM composition. The relationships of maternal adiposity with HM total lipids concentration have recently been reported to be dependent on the lactation stage. As such, in women with overweight and obesity, no difference in the lipid concentration in colostrum was seen; however, lower concentrations were observed in transitional milk and higher concentrations in mature milk [16]. There is also evidence linking maternal BMI and HM leptin, adiponectin, and insulin concentrations, but the findings vary across studies. A systematic review has established that most studies report a positive relationship between maternal BMI and HM leptin concentration, with weaker evidence for HM adiponectin and insulin concentrations [17].

Male and female infants have different growth and neurodevelopmental trajectories, and it is speculated that the dynamics of specific components in HM are a contributing factor. Animal model studies (primate and bovine) have reported that milk composition may differ according to the offspring sex [18,19,20,21]. In humans, limited and conflicting evidence exists regarding sex-specific differences in HM composition [22], with a few studies documenting a higher milk energy content for either male [23,24] or female [25] infants. However, the absence of a difference between HM composition for male and female infants has also been reported [26]. Higher HM leptin concentrations have been reported for female infants [27]. HM leptin and insulin concentrations were also reportedly higher for females born to mothers with obesity [28]. Similarly, mothers of female infants that had gestational diabetes mellitus (GDM), a condition associated with higher adiposity [29,30], had a higher HM adiponectin concentration compared with mothers with male infants and GDM, as well as mothers of infants of both sexes without GDM [31]. Whilst the compositional differences between milk of mothers with and without GDM have been observed, namely for HM adiponectin, insulin, and ghrelin [32], it is not clear if and how these differences persist into the intakes of these components by the infants, as mothers with diabetes and GDM have a shorter breastfeeding duration and may have lower milk production [33,34,35]. Further research is needed to comprehensively validate these differences in concentrations and, most importantly, the intakes of HM components as these have been associated with infant BC [4,36].

The primary aims of this study were: (a) to characterize the longitudinal changes in the concentrations and intakes of HM leptin, adiponectin, insulin, and total lipids; (b) to evaluate the effect of maternal BC on the concentrations and infant intakes of these HM components; (c) to evaluate the relationships between the intakes of HM components and infant BC parameters; and finally, (d) to investigate if there are any infant sex-specific differences in the concentrations and intakes of these HM components.

## 2. Materials and Methods

### 2.1. Study Design

**Figure 1:**
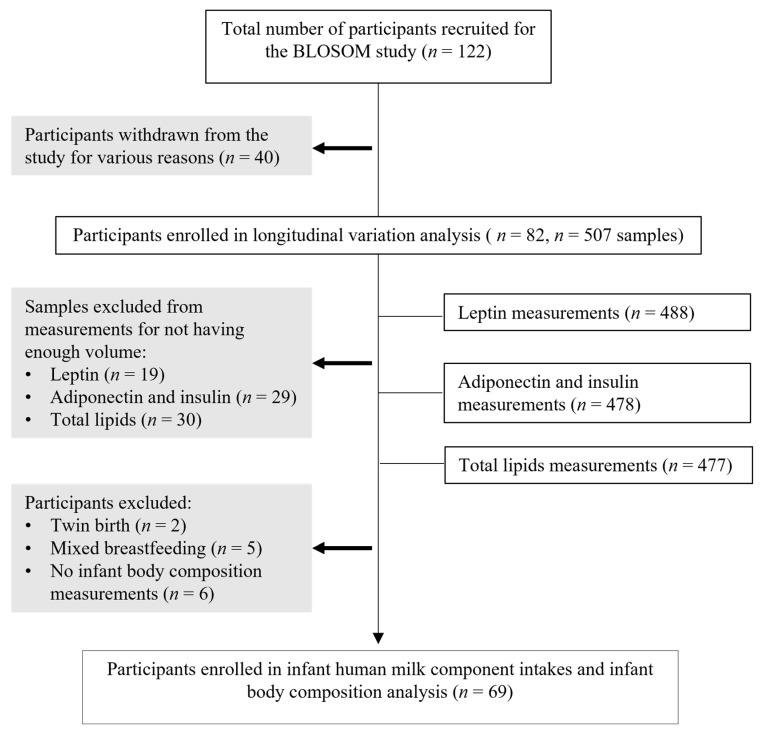
Study flow chart indicating the total number of participants and milk samples analyzed. BLOSOM, Breastfeeding Longitudinal Observational Study of Mothers and kids.

The mother–infant dyads (*n* = 82) were recruited for the BLOSOM (Breastfeeding Longitudinal Observational Study of Mothers and kids) cohort (Figure 1). Women were recruited during the third trimester of pregnancy, from the general population in Perth, Western Australia. The details of the cohort, including the study design, participants’ characteristics, and inclusion and exclusion criteria, have been described previously [37]. Biochemical analyses were performed on all available milk samples (*n* = 501) collected at days 2–5 postpartum and monthly from 1 to 6 months postpartum from women with no major pregnancy complications who intended to breastfeed exclusively for 6 months and to continue breastfeeding up to 12 months. Maternal and infant BC measurements were performed at 3 and 6 months postpartum. All the mothers provided informed written consent to participate in the study, which was approved by the Human Research Ethics Committee at The University of Western Australia (RA/4/20/4023).

### 2.2. Anthropometric and Body Composition Measurements

Anthropometric and BC measurements were performed in mothers and infants at 3 and 6 months postpartum [37]. Electronic scales were used to measure the maternal (±0.1 kg; Seca, Chino, CA, USA) and infant (±2.0 g; Baby Weigh Scales, Medela Inc., McHenry, IL, USA) weight. Height was self-reported by mothers or measured against a calibrated marked wall (accuracy ± 0.1 cm), if unknown. Infant crown to heel length was measured using an infantometer (±1.0 mm; Seca, Chino, CA, USA). Whole body bioimpedance (wrist-to-ankle) of mothers and infants was measured using the ImpediMed SFB7 bioelectrical impedance analyzer (ImpediMed, Brisbane, QLD, Australia), according to the manufacturer’s instructions (mothers) and custom research protocol (infants) [4]. The BC parameters (fat-free mass (FFM), FFM index (FFMI), fat mass (FM), FM index (FM), percentage FM (%FM), and FM-to-FFM ratio (FM/FFM)) were calculated as previously described [4]. BMI-for-age (BMIFA), length-for-age (LFA), weight-for-age (WFA), and weight-for-length (WFL) z scores were calculated using the World Health Organization (WHO) Anthro software v3.2.2 (WHO, Geneva, Switzerland) [38].

### 2.3. Milk Sample Collection

Milk samples were collected by mothers at 2–5 days and monthly from 1 to 6 months postpartum from one breast. Mothers refrained from breastfeeding or expressing milk for at least 2 h before collection. Approximately 20 mL of milk were directly hand-expressed into sterile tubes. The time of sample collection was recorded, and the samples were stored at 4 °C in the participant’s home refrigerator for a maximum of 24 h before being transported on ice to the laboratory. Upon arrival, the samples were aliquoted into 1 mL sterile tubes and stored at −80 °C until further analysis.

### 2.4. Biochemical Analysis

Milk samples for the biochemical analysis (leptin, adiponectin, insulin, and total lipids) were prepared by thawing at room temperature, followed by homogenization using a beads homogenizer. After aliquoting, measurements of HM components were performed in duplicate. Whole HM leptin, adiponectin, and insulin concentrations were analyzed using ELISA kits [39]. Total lipids concentration (%) was determined using the creamatocrit method [40]. The detailed analytical procedures are provided in Supplementary Table S1.

### 2.5. Calculation of Infant Intake of Human Milk Components

At 3 months postpartum (3.07 ± 0.16, range: 2.8–3.9 months), infant 24 h HM intake was measured in-home using the previously described 24 h milk profile protocol [41,42]. Milk intake (g) was converted to volume (mL) using the HM density of 1.03 g/mL [43] and used to calculate the infant HM component intakes at 1, 2, 3, 4, 5, and 6 months of age. The 3-month milk intake represents a typical intake during exclusive breastfeeding [44,45]. The reported average HM intake of 498 g/24 h (483.5 mL/24 h) [43] was used to calculate the infant HM component intakes during the first week postpartum. The mean time of milk sample collection at one week postpartum was 4.1 ± 2.1 days (range: 1–8). 

### 2.6. Statistical Analysis

A statistical analysis was performed using the R Core Team [46]. Standard graphical methods and the Shapiro–Wilk test were used to check for normality of HM components concentrations and intakes, and maternal and infant anthropometrics and BC parameters. Differences between both the maternal and infant BC measurements at 3 and 6 months were analyzed using the Student paired *t*-test, and the Wilcoxon rank-sum test for nonparametric data. A linear mixed models analysis was performed using the lmer function from the lme4 package [47], the significance was calculated using the lmerTest package [48], and a pairwise comparison with post hoc correction was performed using the “emmeans” package [49]. The “ggplot2” [50] and “ggpubr” [51] were used for data visualization. Unless otherwise stated, values are presented as mean  ±  standard deviation (SD). In all the analyses, standard visual diagnostics were performed to assess model assumptions and, when necessary, log transformation of the variables was performed for the HM component concentrations and intakes to meet the statistical test assumptions. Holm’s method was used to control for the family-wise error rate (FWER) when conducting multiple hypothesis testing as appropriate. The *p*-value was considered statistically significant at <0.05.

The times of sample collection were grouped based on the sampling time into four equal intervals: 4:01 AM to 10:00 AM (morning), 10:01 AM to 4:00 PM (afternoon), 4:01 PM to 10:00 PM (evening), and 10:01 PM to 4:01 AM (night). A cumulative average of infant intake of each measured HM component was calculated for the period before each BC measurement to represent long-term exposure, i.e., the cumulative average intake of the HM component at 3 months is the average of infant intakes of the HM component at 1, 2, and 3 months, and the cumulative average intake of the HM component at 6 months is the average of infant intakes of the HM component at 1, 2, 3, 4, 5, and 6 months.

Longitudinal changes in the HM component concentrations from week 1 to 6 months postpartum were analyzed by fitting two linear mixed models. The base model was fitted to investigate the overall longitudinal changes for each response variable (leptin, adiponectin, insulin, and total lipids), with the time postpartum (months) as the explanatory variable and the participant as a random effect. The adjusted model expands the outcomes of the base model by adjusting for the time of sample collection, infant sex, and breastfeeding type at 1 week postpartum (exclusive or mixed breastfeeding). An analysis of variance (ANOVA) was conducted to assess the overall significance of the fixed effects in the linear mixed model with post hoc Holm’s correction. Pairwise comparisons with post hoc Holm’s correction were performed to understand the differences in concentration and intake between the postpartum time points. Longitudinal variations in infant HM component intakes from week 1 to 6 months of age were analyzed using the same models used for longitudinal changes in milk component concentrations by replacing the response variables with leptin intake, adiponectin intake, insulin intake, and total lipids intake. For the missing values of 24 h milk intake (*n* = 21), the mean imputation (mean = 761.5 mL) was employed to calculate the infant HM component intakes.

For the associations between the HM component concentrations and maternal BC parameters (3-month data), a linear mixed model was fitted with the HM component concentrations as the response variable and the maternal BC parameters as the predictors, time postpartum and time of sample collection as the explanatory variables, and the participant as a random effect.

For the associations between the cumulative average intakes of HM components and infant BC parameters, linear mixed models were used with the participant as a random effect. Here, the models were fitted with the predictor, infant age, time of sample collection, and infant sex as explanatory variables.

The effect of infant sex on the concentrations and intakes of HM leptin, adiponectin, insulin, and total lipids was tested while adjusting for time postpartum, time of sample collection, and maternal adiposity (FM/FFM). The model with interaction terms (time postpartum) was also fitted.

## 3. Results

### 3.1. Participants’ Characteristics

In total, data on 82 participants who gave birth to healthy infants and provided milk samples (*n* = 507) were used in the HM composition analyses. For the associations between infant BC measurements and HM component intakes, 13 participants were excluded due to the missing of infant BC measurements at 3 and 6 months (*n* = 6), twin births (*n* = 2), and mixed breastfeeding (*n* = 5). The participant characteristics are presented in Table 1, and the BC parameters are presented in Table 2.

### 3.2. Longitudinal Variations in HM Component Concentrations and Intakes

**Figure 2:**
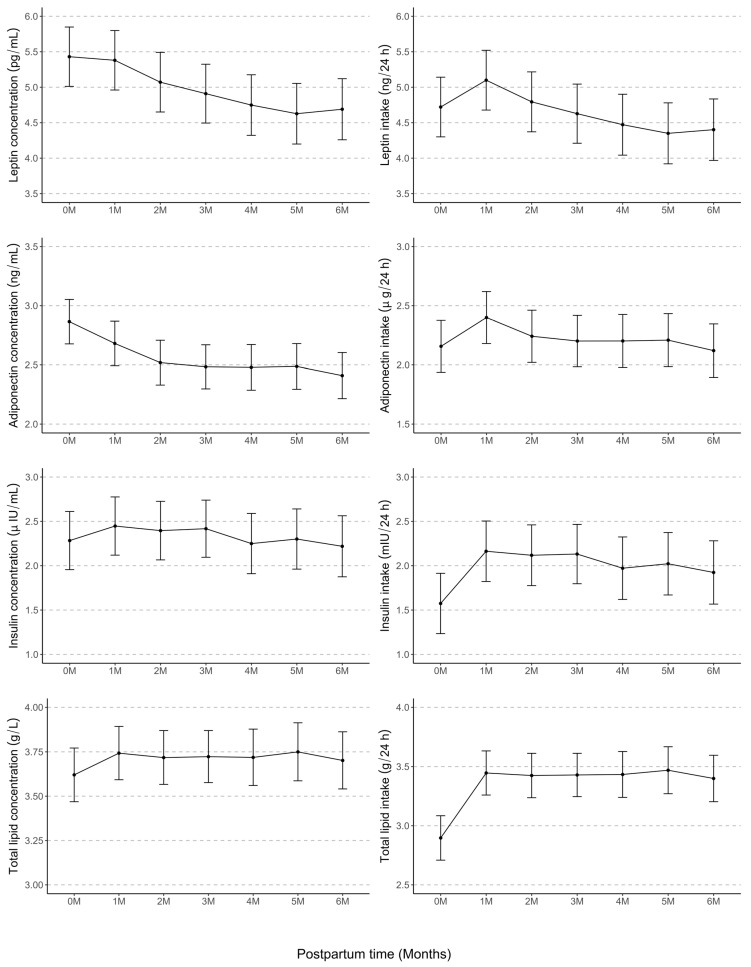
Longitudinal changes in concentrations and 24 h intakes of human milk leptin, adiponectin, insulin, and total lipids from 2–5 days (0M) to 6 months (1M, 2M, 3M, 4M, 5M, and 6M) postpartum. Change in HM components at each time point is presented as estimated mean (black dots) and 95% confidence interval (black error bars). M, month.

HM leptin and adiponectin concentrations decreased significantly from week 1 to month 6 postpartum (*p* < 0.001 for both) (Table 3 and Table 4, Figure 2). Infant intake of leptin and adiponectin increased from 1 week (0M) to 1 month (1M) (leptin: 0.38, 95% CI [0.16, 0.60], *p* < 0.001; adiponectin: 0.24, 95% CI [0.12, 0.36], *p* < 0.001), then remained stable to month 6 postpartum (Table 3 and Table A1, Figure 2).

HM insulin and total lipids concentrations remained consistent over 6 months (insulin: *p* = 0.80; total lipids: *p*  =  1.00; Table 3 and Table 4; Figure 2). HM insulin and total lipids intakes increased from 1 week to 1 month (insulin: 0.59, 95% CI [0.35, 0.83], *p* < 0.001; total lipids: 0.55, 95% CI [0.41, 0.69], *p* < 0.001), then remained stable to month 6 postpartum (Table 4 and Table A1, Figure 2).

### 3.3. Maternal Body Composition and Concentrations and Intakes of Human Milk Components

The associations between maternal BC and the concentrations and infant intakes of HM milk components are presented in Table A2. The concentrations and infant intakes of HM leptin and insulin had positive associations with maternal BMI and all the BC parameters (FFM, FFMI, FM, FMI, %FM, FM/FFM). The adiponectin concentration and intake showed negative associations with the maternal adiposity parameters (concentration: % FM, FM/FFM; intake: FM, FMI, % FM, FM/FFM). The total lipids concentration had positive associations with maternal FFM only (FFM and FFMI), whilst the total lipids intake had no relationship with maternal BC or BMI.

### 3.4. Infant Body Composition and Cumulative Average Intakes of Human Milk Components

The associations between infant BC parameters measured at 3 and 6 months and the cumulative average intakes of HM leptin, adiponectin, insulin, and total lipids at 3 and 6 months postpartum are presented in Table 5. The cumulative average intakes of leptin, adiponectin, and insulin had no relationships with the infant BC parameters, BMI, or z scores (BMIFA, LFA, WFA, and WFL) (Table 5). Both the cumulative average intake of total lipids and infant 24 h HM intake had positive associations with all infant BC parameters, BMI, and z scores, except for LFA, which had no association with the total lipids intake (Table 5).

### 3.5. Human Milk Composition Does Not Differ by Infant Sex

**Figure 3:**
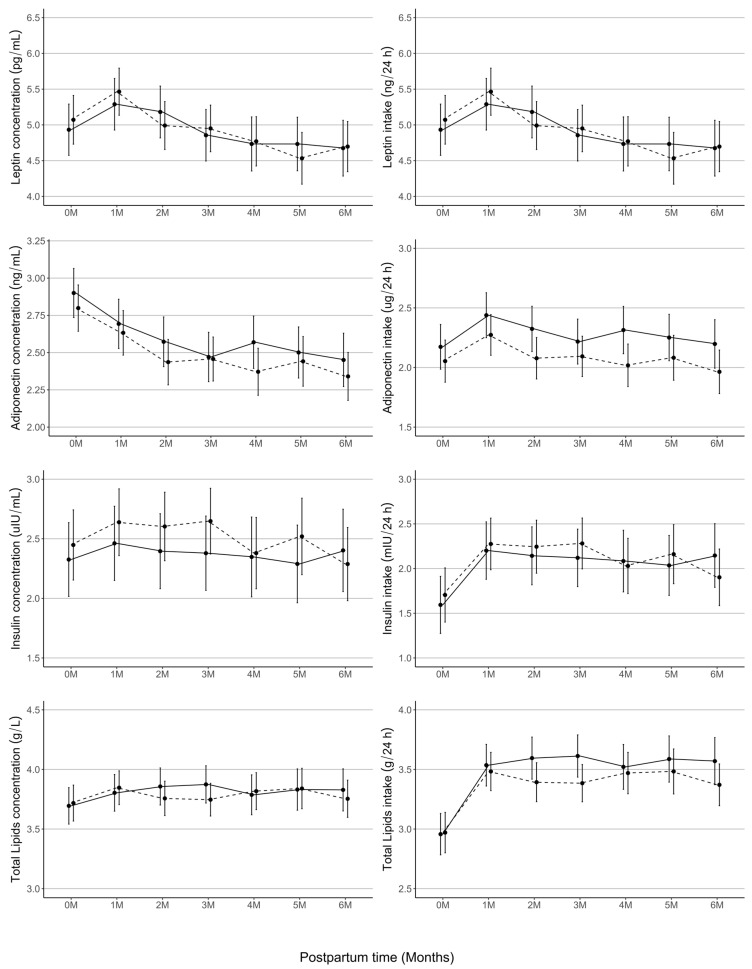
Longitudinal changes in concentrations and 24 h intakes of human milk leptin, adiponectin, insulin, and total lipids from 2–5 days (0M) to 6 months (1M, 2M, 3M, 4M, 5M, and 6M) postpartum. Predicted concentrations and intakes by infant sex over lactation are based on linear mixed-effect models adjusted for postpartum time, maternal adiposity, and time of sample collection. Changes in HM components at each time point are presented as estimated mean (black dots) and 95% confidence intervals (black error bars). Straight line represents males, dotted line represents females. M, month.

There was no significant effect of infant sex on leptin (concentration: −0.16, 95% CI [−0.64, 0.32], *p* = 0.52; intake: −0.14, 95% CI [−0.62, 0.34], *p* = 0.57), adiponectin (concentration: 0.10, 95% CI [−0.12, 0.32], *p* = 0.36; intake: 0.12, 95% CI [−0.13, 0.37], *p* = 0.35), insulin (concentration: −0.12, 95% CI [−0.54, 0.29], *p* = 0.56; intake: −0.11, 95% CI [−0.54, 0.31], *p* = 0.61), and total lipids (concentration: −0.02, 95% CI [−0.23, 0.18], *p* = 0.82; intake: −0.01, 95% CI [−0.25, 0.22], *p* = 0.91) (Figure 3).

## 4. Discussion

We found that the HM leptin and adiponectin concentrations decreased over the first 6 months of lactation, while the infant intakes of HM leptin, adiponectin, insulin, and total lipids increased from 1 week to 1 month and remained stable to 6 months of lactation. Notably, infant 24 h milk intake and total lipids intake were found to associate positively with infant BMI, z scores, FM, and FFM parameters. Importantly, we found no infant sex differences for both concentrations and intakes of all the tested HM components.

In line with other studies, we found that the HM leptin and adiponectin concentrations decreased from 1 week to 3 months and then remained stable [5,52], although some studies have reported the HM leptin concentration to be stable over time [53,54]. Maternal leptin, produced by the placenta and adipose tissue, increases as it plays an important role in pregnancy [55]. In the postpartum period, leptin is transferred from the maternal circulation to the milk [56] and decreases as maternal weight and body composition normalize [57]. Insulin and total lipids on the other hand remained constant [11,58,59,60]. Maternal insulin levels are lower during lactation compared with pregnancy in response to lower blood glucose levels, as the breast demands more energy to produce milk [61]. The process of initiation of milk synthesis occurs within approximately 48 h postpartum in women, and milk production is established by days 8–10 postpartum [62,63], which likely explains the relatively stable HM insulin levels observed in our study. Similarly, lipids do not vary from colostrum to mature milk [64]; however, the fat concentration is well known to exhibit changes over the day, and from before to after in association with breast emptying, whether by breastfeeding or milk expression [39,60,65].

The 24 h milk intake increases rapidly from colostrum to the mature milk stage and stays relatively stable from 1 to 6 months of lactation [43,44,63,66]. However, very few studies have investigated the longitudinal variations in the infant intake of HM components throughout lactation. The only difference we found in our study was an increase in the HM leptin and adiponectin intakes from 1 week to 1 month, after which they remained stable up to 6 months. Infant intakes of HM insulin and leptin also displayed a similar trend (Table A1 and Table A2). This pattern is perhaps not surprising as we used an estimation of 24 h milk intake (498 g/24 h) for 1 week postpartum [43]. Unfortunately, other studies have not systematically measured the HM component intakes after birth, limiting comparisons [36,60,67].

As highlighted previously, the 24 h milk intake is one of the main drivers of infant growth [4,60,68,69]. Indeed, we saw the increased HM intake associated with the increased accrual of infant FM and FFM. In terms of BC development, this is in agreement with a cross-sectional study at 3 months that documented positive relationships of milk intake with infant weight, length, lean body mass (FFM and FFMI), adiposity (FM and FMI), and WAZ [37]. Similarly, two longitudinal studies that spanned the first 12 months of life found 24 h milk intake to be positively associated with infant subcutaneous-abdominal and visceral depths [70], as well as with a larger mid-arm fat area [71].

Furthermore, we found that the increased cumulative average intakes of HM lipids were associated with increased infant FM, FFM, and BMI. Other studies have also found links between lipid intake and growth, with reports of a higher infant intake of HM total fat in infants with high weight gain compared with those with low weight gain [72]. George et al. [73] also reported positive time-dependent relationships between the intakes of multiple individual milk fat globule membrane lipid species and infant height, weight, and head circumference. On the contrary, two longitudinal studies did not find any fat intake relationships with either infant anthropometry [60,74] or skinfold gain [74]. Total HM lipids make up a majority (50%) of the infant’s daily energy intake [75]; therefore, it is counterintuitive that HM lipids intake does not influence infant growth. The discrepancies may arise from the major limitations of the previous studies, including sampling, with only one study [60] calculating fat intake based on samples collected over 24 h.

We have not established any relationships between the intakes of HM metabolic hormones (adiponectin, leptin, and insulin) and infant FM or FFM. Similarly, no relationships with the intakes of these hormones were found with regional fat deposits [70,71]. This is in contrast to the study that reported positive relationships for whole milk leptin [36] and adiponectin [36] intake and infant adiposity parameters, with adiponectin also negatively relating to infant FFM [36]. This is likely due to the different time periods of study, i.e., 1 week to 6 months vs. 3 time points across the first year. Kon et al. [72] also reported higher skim milk leptin and adiponectin intakes in infants with high weight gain compared with low weight gain infants, but no BC was measured. 

Maternal adiposity is increasingly being shown to associate with the concentrations of various HM components. Several studies report inconclusive relationships of maternal adiposity with HM insulin, a lack of relationships with adiponectin, and positive relationships with leptin [17,76] and total lipids [16,77]. We found that higher maternal FM and FFM parameters were associated with higher concentrations of HM leptin and insulin, agreeing with other studies in longitudinal cohorts [36,53,67,78,79]. Whilst our study and others have shown no relationships between adiponectin and maternal BMI [17], we did find lower concentrations of HM adiponectin with higher maternal adiposity (%FM and FM/FFM ratio). This is logical as HM adiponectin concentrations rise with increasing maternal serum adiponectin concentrations [80,81], which in turn are negatively influenced by maternal weight and adiposity [81,82]. These maternal BC relationships were also reflected in the infant intakes of these components, with both HM leptin and insulin intakes associating positively with maternal FM and FFM parameters, and adiponectin intake associating negatively with maternal adiposity.

In contrast to a recent meta-analysis that concluded HM lipids concentration to be higher in mothers with overweight and obesity [16], we found that increased lipids concentration was related to a higher maternal FFM (FFM and FFMI). A recent longitudinal study found that a lower HM lipids concentration is associated with increased maternal lean body mass [83] and another study found higher concentrations in women with overweight compared with those of normal weight at 6 months [67]. Differences in the time (i.e., circadian patterns [39,84]) and method of sampling may in part explain the variation in the reported relationships between concentrations of HM lipids and maternal BC. Contrary to HM metabolic hormones, total lipids intake in our study was not associated with maternal adiposity. However, we analyzed the total lipids and not the lipid composition of HM, which is extensive and known to vary depending on maternal diet, particularly the long-chain polyunsaturated fatty acids [85]. Further studies are needed to determine the intricate relationships between maternal factors, such as diet and adiposity, HM composition, infant milk intake, and the intakes of HM components.

The effect of infant sex on HM components is an intriguing area of research with very limited and conflicting evidence as to whether HM is sex-specific [22]. We found that the concentrations of total lipids (measured at 7 time points), and the actual intakes of lipids (using 24 h milk intake), were not different between sexes. Similarly, Honore et al. [86] found no significant sex-specific differences in the HM macronutrient concentrations, including fat, among mothers of exclusively breastfed 4-month-old infants (*n* = 77). Another five studies also have shown no difference in the lipids concentration [25,26,67,83,87] and only four studies suggested sex differences [88,89,90,91]. One study showed a higher HM lipids concentration in milk for male infants [91] and two studies showed a higher lipids concentration in milk for female infants [88,90]. The fourth study indicated that socio-economic status mediates the relationship between infant sex and HM lipids concentrations in that economically-sufficient mothers produced milk with higher lipids for male infants, while mothers of a lower socio-economic status produced milk with higher lipids for female infants [89]. Likely, the contradictory results are largely due to other confounding factors, methodology, and the timing of milk sampling.

Leptin is the most studied HM adipokine. Our results confirmed that infant sex has no effect on the whole HM leptin concentration [31,67,79,92,93,94,95] and further expanded this to include no difference in infant leptin intakes. Whilst the majority of the literature supports no impact of sex on HM leptin, one study reported a higher HM leptin concentration only for male infants of mothers with overweight [28]. Another showed a higher HM leptin concentration in the milk of mothers of female infants in the UBCS cohort, and no difference in the SPATZ cohort [27], and an analysis of another German population cohort reported higher milk leptin for female infants [96]. Similar to leptin, there is no evidence of the effect of infant sex on HM adiponectin [79,94,95] and insulin [67,79,93,94,95], with the exception of one study reporting higher HM insulin concentrations for male infants of mothers with overweight status [28]. Since the difference was limited to the infants of mothers with higher adiposity, the small sample size of that group (*n* = 37) may have influenced the results.

Despite no sex differences in the HM component intakes or HM intake, we found that, as expected, male infants had a higher FFM and FFMI, and a lower %FM and FM/FFM than females [97,98,99,100,101,102]. However, although there are no sex-specific differences in the HM component concentrations and intakes, due to the presence of sex-specific infant BC differences, it is important to consider infant sex as a confounding factor when investigating relationships between HM components and infant outcomes.

The major limitation of this study is the sampling of HM for the lipid analysis. Ideally, 24 h samples would provide the most accurate measure of total lipids; however, we collected a relatively large volume (20 mL or more), which would offset the low fat in a typical pre-feed sample, and we also accounted for the timing of the sampling in our analysis. Additionally, less milk samples were available for the analysis from 4 months postpartum, which may affect the results.

## 5. Conclusions

The current study expands our knowledge of the longitudinal variation of HM total lipids, leptin, adiponectin, and insulin, and suggests that infant 24 h milk intake and total lipids intake, but not the intakes of metabolic hormones, drive infant growth and fat and lean tissue accrual. Additionally, we found no differences in the concentrations and 24 h intakes of HM total lipids and metabolic hormones in relation to infant sex.

## Figures and Tables

**Table 1:** Participant characteristics ^1^.

Baseline Characteristics *n* = 82	Mean ± SD (Min–Max), or *n* (%)
Maternal age at birth (years)	32.7 ± 5.0 (17.0–46.0)
Parity	
Primiparous	20 (24.4%)
Multiparous	
2	36 (43.9%)
3	23 (28.7%)
4	3 (3%)
Race	
Caucasian	70 (85.4%)
Asian	7 (8.5%)
Other	5 (6.1%)
Infant gestational age (weeks)	39.3 ± 1.2 (36.6–41.2)
Birth weight (kg)	3.5 ± 0.4 (2.4–4.3)
Height (cm)	50.9 ± 2.5 (45.0–59.0)
Mode of delivery	
Vaginal	57 (72%)
Caesarean section	25 (28%)
Infant sex ^2^	
Male	46 (54.8%)
Female	38 (45.2%)
24 h milk intake at 3 months (mL) ^3^	754.5 ± 193.6 (337.9–1304.9)
Breastfeeding mode at 1 week	
Exclusive	75 (91.5%)
Mixed breastfeeding	7 (8.5%)

^1^ Data are presented as mean ± standard deviation (SD), (Min–Max) or *n* (%). ^2^ *n* = 84 (due to twin births). ^3^ *n* = 61.

**Table 2:** Maternal and infant body composition measurements at 3 and 6 months postpartum ^1^.

Maternal Characteristics	3 Months, *n* = 76	6 Months, *n* = 56	*p*-Value
Weight (kg)	71.6 ± 14.6 (47.5–119.8)	70.1 ± 14.9 (47.4–121.6)	0.60 ^2^
BMI (kg/m^2^)	26.1 ± 5.0 (17.6–42.0)	25.8 ± 5.5 (16.2–42.6)	0.57 ^2^
FFM (kg)	45.6 ± 7.5 (33.9–70.9)	44.6 ± 6.7 (33.4–66.5)	0.63 ^2^
FFMI (kg/m^2^)	16.6 ± 2.4 (11.6–24.0)	16.4 ± 2.4 (10.5–22.2)	0.59 ^2^
FM (kg)	26.0 ± 8.9 (13.2–53.9)	25.5 ± 9.1 (12.0–57.6)	0.92 ^2^
FMI (kg/m^2^)	9.5 ± 3.2 (4.8–19.8)	9.4 ± 3.4 (4.6–21.2)	0.91 ^2^
%FM	35.6 ± 5.7 (19.2–53.4)	35.6 ± 5.5 (22.9–49.7)	0.82 ^2^
FM/FFM	0.6 ± 0.1 (0.2–1.1)	0.6 ± 0.1 (0.3–1.0)	0.68 ^3^
**Infant Characteristics**	**3 Months, *n* = 69**	**6 Months, *n* = 51**	
Height (cm)	61.1 ± 2.4 (55.5–67.0)	67.6 ± 2.6 (62.5–74.5)	**<0.001 ^3^**
Weight (kg)	6.2 ± 0.8 (4.1–8.4)	7.8 ± 1.0 (5.4–10.8)	**<0.001 ^3^**
BMI (kg/m^2^)	16.5 ± 1.4 (12.3–19.9)	16.9 ± 1.5 (13.0–21.4)	0.07 ^3^
FFM (kg)	4.6 ± 0.4 (3.8–5.9)	5.4 ± 0.6 (4.1–7.2)	**<0.001 ^3^**
FFMI (kg/m^2^)	15.1 ± 1.1 (13.2–17.6)	16.0 ± 1.4 (13.0–19.4)	**<0.001 ^3^**
FM (kg)	1.6 ± 0.3 (0.8–2.5)	2.3 ± 0.4 (1.3–3.6)	**<0.001 ^3^**
FMI (kg/m^2^)	5.3 ± 0.8 (3.0–7.5)	6.9 ± 1.1 (3.9–9.7)	**<0.001 ^3^**
%FM	25.9 ± 2.3 (18.0–31.1)	29.9 ± 2.2 (22.0–33.7)	**<0.001 ^2^**
FM/FFM	0.4 ± 0.0 (0.2–0.5)	0.4 ± 0.0 (0.3–0.5)	**<0.001 ^2^**
BMIFA	−0.1 ± 0.9 (−3.4–2.0)	−0.2 ± 1.1 (−3.5–2.5)	0.92 ^2^
LFA	0.2 ± 1.1 (−3.1–2.8)	0.4 ± 1.1 (−1.3–3.3)	0.36 ^2^
WFA	0.0 ± 0.9 (−2.8–2.5)	0.1 ± 1.1 (−2.7–2.9)	0.31 ^2^
WFL	−0.1 ± 1.0 (−3.6–2.5)	−0.1 ± 1.1 (−3.5–2.6)	0.55 ^2^

^1^ Data are presented as mean ± standard deviation (SD), (Min-Max). ^2^ Paired *t*-test was used to compare the mean of the normally distributed variables and ^3^ Wilcoxon rank sum test was used for comparison of the mean of non-normally distributed variables at 3 and 6 months of age. BMI, body mass index; BMIFA, BMI-for-age; FFM, fat-free mass; FFMI, fat-free mass index; FM, fat mass; %FM, percentage fat mass; FM/FFM, fat mass to fat-free mass ratio; FMI, fat mass index; LFA, length-for-age; WFA, weight-for-age; WFL, weight-for-length. Bold font indicates a significant difference.

**Table 3:** Concentrations and intakes of human milk components during the first 6 months of lactation ^1^.

Components	2–5 Days *n* = 76	1 Month *n* = 79	2 Months *n* = 75	3 Months *n* = 78	4 Months *n* = 68	5 Months *n* = 59	6 Months *n* = 62	*p*-Value ^2^	*p*-Value ^3^
Concentrations									
Leptin (pg/mL)	485.9 ± 535.7, 294.8 (181.7–573.8)	429.3 ± 421.8, 243.3 (166.1–573.4)	329.9 ± 333.2, 200.0 (101.7–466.3)	342.1 ± 415.6, 175.4 (78.9–477.0)	280.0 ± 313.3, 116.3 (59.9–421.7)	254.9 ± 285.3, 132.0 (62.5–369.3)	250.6 ± 244.7, 152.9 (61.5–411.7)	**<0.001**	**<0.001**
Adiponectin (ng/mL)	22.1 ± 19.2, 16.6 (12.8–22.8)	15.2 ± 6.2, 14.4 (11.0–18.5)	12.7 ± 4.7, 11.9 (9.5–15.6)	12.3 ± 6.5, 11.3 (8.6–14.4)	14.3 ± 16.9, 10.6 (8.0–14.3)	13.2 ± 8.1, 11.2 (8.8–15.7)	11.6 ± 5.5, 10.0 (7.7–14.6)	**<0.001**	**<0.001**
Insulin (µIU/mL)	18.8 ± 32.5, 13.7 (5.3–20.2)	15.6 ± 9.4, 15.0 (9.0–20.4)	15.5 ± 10.8, 14.2 (8.9–21.0)	14.9 ± 11.0, 12.6 (6.0–20.4)	13.0 ± 12.0, 9.8 (6.1–18.1)	15.5 ± 11.8, 13.3 (5.9–23.0)	13.6 ± 16.2, 9.8 (3.6–16.1)	**0.03**	0.8
Total lipids (g/L)	43.4 ± 14.9, 42.7 (31.7–52.0)	48.1 ± 16.6, 44.5 (37.7–54.9)	48.4 ± 16.3, 45.5 (37.3–57.7)	50.8 ± 22.8, 48.4 (33.6–60.8)	49.5 ± 20.9, 49.5 (34.4–61.7)	51.7 ± 22.7, 49.1 (37.0–64.1)	47.5 ± 20.5, 44.1 (34.0–58.1)	0.48	1.0
Intakes									
Leptin (ng/24 h)	234.9 ± 259.0, 142.5 (87.8–277.4)	309.5 ± 295.5, 182.0 (112.9–409.8)	238.9 ± 237.6, 146.4 (85.5–322.0)	243.4 ± 290.8, 111.9 (60.3–363.0)	196.6 ± 208.6, 112.7 (57.1–295.2)	182.9 ± 199.2, 112.6 (51.6–259.4)	174.5 ± 168.1, 111.0 (48.0–261.0)	**<0.001**	**<0.001**
Adiponectin (µg/24 h)	10.7 ± 9.3, 8.0 (6.2–11.0)	11.6 ± 5.8, 10.0 (7.4–14.3)	9.8 ± 4.6, 9.5 (6.6–12.2)	9.5 ± 6.1, 8.6 (5.3–11.3)	11.3 ± 15.2, 8.6 (5.5–10.8)	10.2 ± 7.0, 8.6 (6.6–12.0)	8.8 ± 5.2, 7.2 (5.3–10.8)	**<0.001**	**0.001**
Insulin (mIU/24 h)	9.1 ± 15.7, 6.6 (2.6–9.8)	11.7 ± 7.6, 10.4 (6.3–15.3)	11.5 ± 8.1, 10.4 (6.1–15.8)	10.9 ± 8.1, 9.8 (4.4–13.9)	10.0 ± 9.7, 7.7 (4.6–12.5)	11.7 ± 8.8, 9.3 (4.2–18.1)	9.8 ± 12.1, 6.3 (2.5–11.8)	**<0.001**	**0.001**
Total lipids (g/24 h)	21.0 ± 7.2, 20.6 (15.3–25.2)	36.5 ± 16.0, 32.1 (25.1–43.3)	36.7 ± 15.5, 35.9 (26.6–42.8)	38.7 ± 20.3, 34.7 (23.8–45.7)	38.1 ± 17.8, 33.5 (25.3–46.8)	40.6 ± 21.0, 39.1 (29.5–46.0)	35.3 ± 16.8, 35.3 (22.6–44.3)	**<0.001**	**<0.001**

^1^ Data are presented as mean ± standard deviation (SD) and median (25th—75th). ^2^ The *p*-values represent the significance levels obtained from the analysis of variance (ANOVA) for the base models with Holm’s correction. ^3^ The *p*-values represent the significance levels obtained from the analysis of variance (ANOVA) for the base models with Holm’s correction (adjusted for maternal FM/FFM, infant sex, time of sample collection, and breastfeeding type at 1 week postpartum). Bold font indicates a significant difference.

**Table 4:** Longitudinal changes in concentrations and intakes of human milk components ^1^.

Predictors	Leptin				Adiponectin				Insulin				Total Lipids			
	Base Model		Adjusted Model		Base Model		Adjusted Model		Base Model		Adjusted Model		Base Model		Adjusted Model	
	β (SE)	*p*-Value ^2^	β (SE)	*p*-Value ^3^	β (SE)	*p*-Value ^2^	β (SE)	*p*-Value ^3^	β (SE)	*p*-Value ^2^	β (SE)	*p*-Value ^3^	β (SE)	*p*-Value ^2^	β (SE)	*p*-Value ^3^
Concentrations																
Intercept	5.76 (0.13)		3.87 (0.41)		2.90 (0.05)		3.13 (0.18)		2.45 (0.10)		1.42 (0.30)		3.71 (0.05)		3.74 (0.13)	
Month 1	−0.09 (0.11)	0.40	−0.05 (0.11)	1.0	−0.26 (0.06)	**<0.001**	−0.18 (0.06)	**0.007**	0.08 (0.12)	1.0	0.16 (0.12)	1.0	0.11 (0.06)	0.37	0.12 (0.07)	0.8
Month 2	−0.42 (0.11)	**<0.001**	−0.36 (0.12)	**0.01**	−0.42 (0.06)	**<0.001**	−0.35 (0.06)	**<0.001**	−0.01 (0.13)	1.0	0.11 (0.12)	1.0	0.12 (0.06)	0.37	0.10 (0.07)	1.0
Month 3	−0.64 (0.11)	**<0.001**	−0.52 (0.11)	**<0.001**	−0.48 (0.06)	**<0.001**	−0.38 (0.06)	**<0.001**	−0.06 (0.12)	1.0	0.13 (0.12)	1.0	0.12 (0.06)	0.37	0.10 (0.07)	1.0
Month 4	−0.75 (0.11)	**<0.001**	−0.68 (0.12)	**<0.001**	−0.47 (0.06)	**<0.001**	−0.39 (0.06)	**<0.001**	−0.21 (0.13)	0.4	−0.03 (0.13)	1.0	0.10 (0.07)	0.37	0.10 (0.07)	1.0
Month 5	−0.88 (0.12)	**<0.001**	−0.80 (0.12)	**<0.001**	−0.45 (0.06)	**<0.001**	−0.38 (0.06)	**<0.001**	−0.10 (0.13)	1.0	0.02 (0.13)	1.0	0.12 (0.07)	0.37	0.13 (0.08)	0.8
Month 6	−0.81 (0.11)	**<0.001**	−0.74 (0.12)	**<0.001**	−0.54 (0.06)	**<0.001**	−0.46 (0.06)	**<0.001**	−0.33 (0.13)	0.07	−0.06 (0.13)	1.0	0.06 (0.07)	0.37	0.08 (0.07)	1.0
Intakes																
Intercept	5.02 (0.13)	**<0.001**	3.22 (0.41)	**<0.001**	2.16 (0.06)	**<0.001**	2.49 (0.21)	**<0.001**	1.71 (0.11)	**<0.001**	0.78 (0.31)	0.073	2.96 (0.06)	**<0.001**	3.10 (0.17)	**<0.001**
Month 1	0.34 (0.11)	**0.005**	0.38 (0.11)	**0.010**	0.18 (0.06)	**0.015**	0.24 (0.06)	**<0.001**	0.51 (0.12)	**<0.001**	0.59 (0.12)	**<0.001**	0.54 (0.07)	**<0.001**	0.55 (0.07)	**<0.001**
Month 2	0.02 (0.11)	0.854	0.07 (0.12)	1.00	0.02 (0.06)	1.00	0.08 (0.06)	1.00	0.43 (0.13)	**0.004**	0.54 (0.13)	**<0.001**	0.55 (0.07)	**<0.001**	0.53 (0.07)	**<0.001**
Month 3	−0.21 (0.11)	0.098	−0.09 (0.11)	1.00	−0.05 (0.06)	1.00	0.04 (0.06)	1.00	0.37 (0.12)	0.011	0.56 (0.12)	**<0.001**	0.55 (0.07)	**<0.001**	0.53 (0.07)	**<0.001**
Month 4	−0.31 (0.11)	**0.018**	−0.25 (0.12)	0.32	−0.03 (0.06)	1.00	0.05 (0.06)	1.00	0.22 (0.13)	0.18	0.40 (0.13)	**0.018**	0.54 (0.07)	**<0.001**	0.54 (0.08)	**<0.001**
Month 5	−0.44 (0.12)	**0.001**	−0.37 (0.12)	0.026	−0.01 (0.06)	1.00	0.05 (0.07)	1.00	0.34 (0.13)	**0.03**	0.45 (0.13)	**0.006**	0.57 (0.07)	**<0.001**	0.57 (0.08)	**<0.001**
Month 6	−0.38 (0.12)	**0.004**	−0.32 (0.13)	0.10	−0.11 (0.06)	0.34	−0.04 (0.07)	1.00	0.09 (0.13)	0.51	0.35 (0.14)	0.06	0.49 (0.07)	**<0.001**	0.50 (0.08)	**<0.001**

^1^ Data on HM component concentrations and intakes at different lactation stages from 1 week (intercept) to 6 months postpartum for the base model and the adjusted model. Differences are shown as parameter estimates (β) and standard errors (SE). ^2^ *p*-value indicates a significant difference from the base model with Holm’s correction. ^3^ *p*-value indicates a significant difference from the model (adjusted for maternal BMI, infant sex, time of sample collection, breastfeeding type at 1 week postpartum) with Holm’s correction. Bold font indicates a significant difference.

**Table 5:** Associations between infant body composition measurements and cumulative average intakes of HM components ^1^.

Predictor	Slope (SE)	95% CI	*p*-Value ^2^	*p*-Value ^3^
BMI (kg/m^2^)				
Leptin (ng/24 h)	−0.0009 (0.0006)	−0.0020–0.0003	0.146	0.019
Adiponectin (µg/24 h)	0.0300 (0.0247)	−0.0191–0.0791	0.229	**0.042 ^4^**
Insulin (mIU/24 h)	−0. 0019 (0.0222)	−0.0461–0.0422	0.931	**0.027 ^4^**
Total lipids (g/24 h)	0.0321 (0.0103)	0.0116–0.0526	**0.002**	0.105
Milk intake (mL/24 h)	0.0028 (0.0008)	0.0012–0.0044	**0.001**	0.095
FFM (kg)				
Leptin (ng/24 h)	−0.0003 (0.0002)	−0.0007–0.0001	0.158	**<0.001 ^4^**
Adiponectin (µg/24 h)	0.0091 (0.0081)	−0.0069–0.0251	0.262	**<0.001 ^4^**
Insulin (mIU/24 h)	0.0011 (0.0072)	−0.0133–0.0155	0.878	**<0.001 ^4^**
Total lipids (g/24 h)	0.0077 (0.0034)	0.0008–0.0145	**0.028**	**<0.001 ^4^**
Milk intake (mL/24 h)	0.0010 (0.0003)	0.0004–0.0015	**0.002**	**<0.001 ^4^**
FFMI (kg/m^2^)				
Leptin (ng/24 h)	−0.0007 (0.0005)	−0.0016–0.0002	0.107	**<0.001 ^4^**
Adiponectin (µg/24 h)	0.0283 (0.0189)	−0.0092–0.0659	0.137	**<0.001 ^4^**
Insulin (mIU/24 h)	0.0009 (0.0171)	−0.0330–0.0349	0.957	**<0.001 ^4^**
Total lipids (g/24 h)	0.0236 (0.0080)	0.0076–0.0395	**0.004**	**<0.001 ^4^**
Milk intake (mL/24 h)	0.0021 (0.0006)	0.0008–0.0034	**0.001**	**<0.001 ^4^**
FM (kg)				
Leptin (ng/24 h)	−0.0002 (0.0002)	−0.0006–0.0001	0.171	0.168
Adiponectin (µg/24 h)	0.0055 (0.0067)	−0.0079–0.0189	0.416	0.230
Insulin (mIU/24 h)	−0.0005 (0.0060)	−0.0125–0.0115	0.937	0.193
Total lipids (g/24 h)	0.0072 (0.0028)	0.0016–0.0129	**0.013**	0.415
Milk intake (mL/24 h)	0.0008 (0.0002)	0.0004–0.0013	**0.001**	0.466
FMI (kg/m^2^)				
Leptin (ng/24 h)	−0.0007 (0.0005)	−0.0016–0.0002	0.128	0.403
Adiponectin (µg/24 h)	0.0159 (0.0182)	−0.0203–0.0520	0.386	0.521
Insulin (mIU/24 h)	−0.0037 (0.0163)	−0.0361–0.0287	0.820	0.461
Total lipids (g/24 h)	0.0219 (0.0076)	0.0067–0.0370	**0.005**	0.882
Milk intake (mL/24 h)	0.0023 (0.0006)	0.0010–0.0036	**0.001**	0.911
%FM				
Leptin (ng/24 h)	−0.0016 (0.0010)	−0.0036–0.0004	0.123	0.055
Adiponectin (µg/24 h)	0.0305 (0.0415)	−0.0519–0.1129	0.463	**0.039 ^5^**
Insulin (mIU/24 h)	0.0100 (0.0370)	−0.0636–0.0836	0.788	0.061
Total lipids (g/24 h)	0.0540 (0.0172)	0.0198–0.0882	**0.002**	**0.005 ^5^**
Milk intake (mL/24 h)	0.0055 (0.0014)	0.0027–0.0083	**<0.001**	**0.004 ^5^**
FM/FFM				
Leptin (ng/24 h)	−0.00003 (0.00002)	−0.00007–0.00001	0.136	**0.039 ^5^**
Adiponectin (µg/24 h)	0.0006 (0.0008)	−0.0010–0.0021	0.455	**0.028 ^5^**
Insulin (mIU/24 h)	0.0002 (0.0007)	−0.0012–0.0016	0.783	**0.045 ^5^**
Total lipids (g/24 h)	0.0010 (0.0003)	0.0004–0.0017	**0.002**	**0.003 ^5^**
Milk intake (mL/24 h)	0.00010 (0.00003)	0.00005–0.00015	**<0.001**	**0.003 ^5^**
BMIFA				
Leptin (ng/24 h)	−0.0006 (0.0004)	−0.0014–0.0002	0.151	0.460
Adiponectin (µg/24 h)	0.0221 (0.0175)	−0.0126–0.0569	0.209	0.660
Insulin (mIU/24 h)	−0.0013 (0.0157)	−0.0325–0.0300	0.936	0.507
Total lipids (g/24 h)	0.0234 (0.0073)	0.0090–0.0378	**0.002**	0.941
Milk intake (mL/24 h)	0.0021 (0.0006)	0.0010–0.0032	**<0.001**	0.941
LFA				
Leptin (ng/24 h)	−0.0002 (0.0005)	−0.0012–0.0008	0.711	0.490
Adiponectin (µg/24 h)	0.0107 (0.0208)	−0.0306–0.0520	0.609	0.566
Insulin (mIU/24 h)	0.0206 (0.0184)	−0.0158–0.0571	0.264	0.377
Total lipids (g/24 h)	0.0118 (0.0090)	−0.0060–0.0296	0.190	0.718
Milk intake (mL/24 h)	0.0016 (0.0007)	0.0002–0.0030	**0.030**	0.830
WFA				
Leptin (ng/24 h)	−0.0006 (0.0004)	−0.0014–0.0002	0.148	0.354
Adiponectin (µg/24 h)	0.0087 (0.0160)	−0.0231–0.0405	0.588	0.422
Insulin (mIU/24 h)	−0.0025 (0.0142)	−0.0308–0.0258	0.861	0.393
Total lipids (g/24 h)	0.0155 (0.0067)	0.0023–0.0288	**0.022**	0.649
Milk intake (mL/24 h)	0.0023 (0.0006)	0.0011–0.0036	**<0.001**	0.862
WFL				
Leptin (ng/24 h)	−0.0006 (0.0004)	−0.0014–0.0003	0.184	0.606
Adiponectin (µg/24 h)	0.0261 (0.0182)	−0.0100–0.0622	0.155	0.858
Insulin (mIU/24 h)	0.0006 (0.0165)	−0.0321–0.0332	0.973	0.641
Total lipids (g/24 h)	0.0244 (0.0077)	0.0092–0.0396	**0.002**	0.744
Milk intake (mL/24 h)	0.0019 (0.0006)	0.0008–0.0031	**0.001**	0.803

^1^ Data are parameter estimate ± standard error (SE), effects of predictors taken from linear mixed models that accounted for the time of sample collection, infant age, and sex. ^2^ *p*-values indicate a significant effect of the predictor on infant body composition parameters. ^3^ *p*-values indicate a significant effect of infant sex on infant body composition parameters (^4^ higher fat-free mass and ^5^ lower adiposity parameters in male infants). BMI, body mass index; BMIFA, BMI-for-age; FFM, fat-free mass; FFMI, fat-free mass index; FM, fat mass; %FM, percentage fat mass; FM/FFM, fat mass to fat-free mass ratio; FMI, fat mass index; LFA, length-for-age; WFA, weight-for-age; WFL, weight-for-length. Bold font indicates a significant association.

## Data Availability

Restrictions apply to the availability of some, or all the data generated or analyzed during this study. The corresponding author will on request detail the restrictions and any conditions under which access to some data may be provided.

## Supplementary Materials

The following supporting information can be downloaded at: https://www.mdpi.com/article/10.3390/nu16162739/s1, Supplementary Table S1: Biochemical analyses of human milk components.

## Appendix A

**Table A1:** Pairwise comparison of human milk composition variation from week 1 to 6 months postpartum ^1^.

Contrast	Estimate	SE	*df*	*t*-Ratio	*p*-Value	Estimate	SE	*df*	*t*-Ratio	*p*-Value
Leptin concentration, (pg/mL)						Leptin intake, (ng/24 h)				
0M–1M	0.05	0.11	359.34	0.45	1.00	−0.35	0.11	359.37	−3.09	0.03
0M–2M	0.36	0.12	359.10	3.11	**0.02**	−0.04	0.12	359.13	−0.38	0.70
0M–3M	0.52	0.11	358.42	4.60	**0.00**	0.12	0.11	358.45	1.06	0.70
0M–4M	0.68	0.12	360.79	5.68	**0.00**	0.28	0.12	360.83	2.29	0.20
0M–5M	0.80	0.12	361.02	6.62	**0.00**	0.40	0.12	361.07	3.26	**0.02**
0M–6M	0.74	0.12	362.62	5.98	**0.00**	0.35	0.13	362.68	2.78	0.07
1M–2M	0.31	0.11	359.07	2.73	0.07	0.31	0.11	359.10	2.68	0.09
1M–3M	0.47	0.11	358.72	4.23	**0.00**	0.47	0.11	358.74	4.21	**0.00**
1M–4M	0.63	0.12	358.34	5.44	**0.00**	0.63	0.12	358.37	5.35	**0.00**
1M–5M	0.75	0.12	359.46	6.37	**0.00**	0.75	0.12	359.49	6.28	**0.00**
1M–6M	0.69	0.12	359.13	5.81	**0.00**	0.70	0.12	359.16	5.81	**0.00**
2M–3M	0.16	0.11	358.98	1.43	0.93	0.17	0.11	359.00	1.45	0.70
2M–4M	0.32	0.12	359.52	2.71	0.07	0.32	0.12	359.56	2.68	0.09
2M–5M	0.44	0.12	359.68	3.67	**0.00**	0.44	0.12	359.72	3.63	**0.01**
2M–6M	0.38	0.12	360.93	3.11	**0.02**	0.39	0.12	360.98	3.17	**0.02**
3M–4M	0.16	0.12	360.43	1.40	0.93	0.16	0.12	360.47	1.34	0.70
3M–5M	0.28	0.12	360.60	2.39	0.14	0.28	0.12	360.65	2.32	0.20
3M–6M	0.22	0.12	360.82	1.84	0.47	0.23	0.12	360.87	1.87	0.50
4M–5M	0.12	0.12	358.84	1.00	1.00	0.12	0.12	358.87	0.99	0.70
4M–6M	0.06	0.12	359.80	0.48	1.00	0.07	0.12	359.83	0.56	0.70
5M–6M	−0.06	0.13	359.82	−0.50	1.00	−0.05	0.13	359.86	−0.41	0.70
Adiponectin concentration, (ng/mL)						Adiponectin intake, (µg/24 h)				
0M–1M	0.18	0.06	355.19	3.27	**0.01**	−0.22	0.06	354.51	−3.55	**0.01**
0M–2M	0.35	0.06	354.96	5.97	**0.00**	−0.06	0.06	354.28	−0.91	1.00
0M–3M	0.38	0.06	354.19	6.70	**0.00**	−0.02	0.06	353.63	−0.27	1.00
0M–4M	0.39	0.06	357.03	6.41	**0.00**	−0.02	0.06	356.03	−0.26	1.00
0M–5M	0.38	0.06	358.56	6.22	**0.00**	−0.02	0.07	357.37	−0.36	1.00
0M–6M	0.46	0.06	360.72	7.34	**0.00**	0.06	0.07	359.20	0.97	1.00
1M–2M	0.16	0.06	354.93	2.85	0.05	0.16	0.06	354.28	2.59	0.16
1M–3M	0.20	0.06	354.39	3.54	**0.01**	0.20	0.06	353.84	3.31	**0.02**
1M–4M	0.20	0.06	354.01	3.46	**0.01**	0.20	0.06	353.48	3.16	**0.03**
1M–5M	0.19	0.06	356.42	3.27	**0.01**	0.19	0.06	355.55	3.00	0.05
1M–6M	0.27	0.06	356.27	4.55	**0.00**	0.28	0.06	355.43	4.36	**0.00**
2M–3M	0.04	0.06	354.75	0.62	0.95	0.04	0.06	354.13	0.65	1.00
2M–4M	0.04	0.06	355.50	0.66	0.95	0.04	0.06	354.73	0.62	1.00
2M–5M	0.03	0.06	356.74	0.53	0.95	0.03	0.07	355.79	0.50	1.00
2M–6M	0.11	0.06	358.50	1.78	0.76	0.12	0.07	357.30	1.84	1.00
3M–4M	0.00	0.06	356.60	0.07	0.95	0.00	0.06	355.68	0.00	1.00
3M–5M	0.00	0.06	357.71	−0.06	0.95	−0.01	0.06	356.65	−0.12	1.00
3M–6M	0.07	0.06	358.19	1.23	0.95	0.08	0.06	357.06	1.26	1.00
4M–5M	−0.01	0.06	355.43	−0.12	0.95	−0.01	0.07	354.69	−0.11	1.00
4M–6M	0.07	0.06	356.87	1.12	0.95	0.08	0.07	355.89	1.22	1.00
5M–6M	0.08	0.06	355.89	1.23	0.95	0.09	0.07	355.03	1.31	1.00
Insulin concentration, (µIU/mL)						Insulin intake, (mIU/24 h)				
0M–1M	−0.16	0.12	357.68	−1.36	0.89	−0.56	0.12	357.47	−4.55	**0.00**
0M–2M	−0.11	0.12	357.56	−0.91	0.89	−0.52	0.13	357.33	−4.06	**0.00**
0M–3M	−0.13	0.12	356.38	−1.10	0.89	−0.53	0.12	356.18	−4.25	**0.00**
0M–4M	0.03	0.13	360.72	0.26	0.89	−0.37	0.13	360.40	−2.80	0.09
0M–5M	−0.02	0.13	362.82	−0.13	0.89	−0.42	0.13	362.46	−3.16	**0.03**
0M–6M	0.06	0.13	366.14	0.49	0.89	−0.32	0.14	365.69	−2.37	0.29
1M–2M	0.05	0.12	357.35	0.43	0.89	0.05	0.12	357.14	0.37	0.91
1M–3M	0.03	0.12	356.38	0.25	0.89	0.03	0.12	356.21	0.26	0.91
1M–4M	0.20	0.12	356.04	1.59	0.89	0.19	0.13	355.86	1.50	0.91
1M–5M	0.15	0.13	359.63	1.16	0.89	0.14	0.13	359.36	1.09	0.91
1M–6M	0.23	0.13	359.34	1.79	0.89	0.24	0.13	359.08	1.84	0.91
2M–3M	−0.02	0.12	357.05	−0.18	0.89	−0.01	0.12	356.85	−0.11	0.91
2M–4M	0.15	0.13	358.43	1.14	0.89	0.15	0.13	358.17	1.12	0.91
2M–5M	0.09	0.13	360.27	0.73	0.89	0.10	0.13	359.96	0.72	0.91
2M–6M	0.18	0.13	362.84	1.35	0.89	0.19	0.13	362.47	1.44	0.91
3M–4M	0.17	0.12	359.95	1.36	0.89	0.16	0.13	359.66	1.26	0.91
3M–5M	0.12	0.13	361.49	0.92	0.89	0.11	0.13	361.18	0.85	0.91
3M–6M	0.20	0.13	362.19	1.55	0.89	0.21	0.13	361.85	1.59	0.91
4M–5M	−0.05	0.13	358.20	−0.39	0.89	−0.05	0.13	357.96	−0.38	0.91
4M–6M	0.03	0.13	360.52	0.23	0.89	0.05	0.14	360.20	0.35	0.91
5M–6M	0.08	0.13	359.26	0.61	0.89	0.10	0.14	358.96	0.71	0.91
Total lipid concentration, (g/L)						Total lipid intake, (g/24 h)				
0M–1M	−0.12	0.07	360.59	−1.80	0.99	−0.52	0.07	355.64	−7.39	**0.00**
0M–2M	−0.10	0.07	359.91	−1.40	0.99	−0.50	0.07	354.85	−6.93	**0.00**
0M–3M	−0.10	0.07	358.98	−1.49	0.99	−0.50	0.07	354.07	−7.08	**0.00**
0M–4M	−0.10	0.07	366.35	−1.35	0.99	−0.51	0.08	359.21	−6.75	**0.00**
0M–5M	−0.13	0.08	368.71	−1.72	0.99	−0.54	0.08	360.11	−6.98	**0.00**
0M–6M	−0.08	0.07	371.24	−1.09	0.99	−0.47	0.08	362.14	−6.15	**0.00**
1M–2M	0.02	0.07	357.98	0.37	0.99	0.02	0.07	353.97	0.31	0.96
1M–3M	0.02	0.07	357.50	0.29	0.99	0.02	0.07	353.66	0.24	0.96
1M–4M	0.02	0.07	356.34	0.34	0.99	0.01	0.07	352.44	0.17	0.96
1M–5M	−0.01	0.07	365.06	−0.10	0.99	−0.02	0.08	357.74	−0.30	0.96
1M–6M	0.04	0.07	361.09	0.57	0.99	0.05	0.07	355.34	0.63	0.96
2M–3M	−0.01	0.07	357.20	−0.08	0.99	0.00	0.07	353.11	−0.07	0.96
2M–4M	0.00	0.07	360.98	−0.02	0.99	−0.01	0.07	355.41	−0.12	0.96
2M–5M	−0.03	0.07	363.62	−0.43	0.99	−0.04	0.08	356.47	−0.58	0.96
2M–6M	0.02	0.07	365.81	0.22	0.99	0.02	0.08	358.31	0.33	0.96
3M–4M	0.00	0.07	361.74	0.06	0.99	0.00	0.07	356.22	−0.05	0.96
3M–5M	−0.03	0.07	364.64	−0.37	0.99	−0.04	0.08	357.39	−0.52	0.96
3M–6M	0.02	0.07	363.85	0.30	0.99	0.03	0.07	357.19	0.40	0.96
4M–5M	−0.03	0.08	363.48	−0.41	0.99	−0.04	0.08	356.49	−0.46	0.96
4M–6M	0.02	0.07	363.57	0.23	0.99	0.03	0.08	356.61	0.44	0.96
5M–6M	0.05	0.08	365.59	0.62	0.99	0.07	0.08	357.48	0.87	0.96

^1^ Data are presented as the parameter estimate, standard error (SE), degree of freedom (*df*), *t*-ratio, and *p*-value of the pair comparison made between each pair of groups (time postpartum; M0 to M6) with post hoc Holm’s correction. Each pairwise comparison contrasts the difference in means (or proportions) between two specific groups. Significant differences were indicated at *p* < 0.05 (bold font). M, month.

**Table A2:** Associations between maternal body composition and human milk component concentrations and infant intakes ^1^.

Maternal Predictor	Slope (SE)	95% CI	*p*-Value ^2^	Slope (SE)	95% CI	*p*-Value ^2^
	Leptin (pg/mL)			Leptin intake (ng/24 h)		
BMI (kg/m^2^)	0.12 (0.02)	0.09–0.15	**<0.001**	0.11 (0.02)	0.08–0.15	**<0.001**
FFM (kg)	0.05 (0.01)	0.02–0.07	**0.001**	0.05 (0.01)	0.02–0.07	**0.001**
FFMI (kg/m^2^)	0.20 (0.04)	0.12–0.28	**<0.001**	0.19 (0.04)	0.12–0.27	**<0.001**
FM (kg)	0.07 (0.01)	0.05–0.09	**<0.001**	0.06 (0.01)	0.04–0.08	**<0.001**
FMI (kg/m^2^)	0.20 (0.03)	0.15–0.25	**<0.001**	0.19 (0.03)	0.14–0.24	**<0.001**
% FM	0.09 (0.02)	0.06–0.12	**<0.001**	0.08 (0.02)	0.05–0.12	**<0.001**
FM/FFM	3.55 (0.65)	2.28–4.8	**<0.001**	3.26 (0.64)	2.00–4.53	**<0.001**
	Adiponectin (ng/mL)			Adiponectin (µg/24 h)		
BMI (kg/m^2^)	−0.01 (0.01)	−0.02–0.01	0.408	−0.01 (0.01)	−0.03–0.01	0.210
FFM (kg)	0.01 (0.01)	−0.00–0.02	0.251	0.01 (0.01)	−0.01–0.02	0.461
FFMI (kg/m^2^)	0.01 (0.02)	−0.02–0.05	0.456	0.01 (0.02)	−0.03–0.05	0.719
FM (kg)	−0.01 (0.00)	−0.02–0.00	0.111	−0.01 (0.01)	−0.02–−0.00	**0.044**
FMI (kg/m^2^)	−0.02 (0.01)	−0.05–0.00	0.073	−0.04 (0.02)	−0.07–−0.00	**0.028**
% FM	−0.02 (0.01)	−0.03–−0.01	**0.004**	−0.03 (0.01)	−0.05–−0.01	**0.001**
FM/FFM	−0.76 (0.28)	−1.30–−0.21	**0.007**	−1.05 (0.33)	−1.71–−0.39	**0.002**
	Insulin (µIU/mL)			Insulin intake, (mIU/24 h)		
BMI (kg/m^2^)	0.05 (0.01)	0.03–0.08	**<0.001**	0.05 (0.01)	0.02–0.07	**<0.001**
FFM (kg)	0.02 (0.01)	0.01–0.04	**0.009**	0.02 (0.01)	0.00–0.04	**0.015**
FFMI (kg/m^2^)	0.09 (0.03)	0.04–0.15	**0.001**	0.09 (0.03)	0.03–0.14	**0.002**
FM (kg)	0.03 (0.01)	0.01–0.04	**<0.001**	0.02 (0.01)	0.01–0.04	**0.001**
FMI (kg/m^2^)	0.08 (0.02)	0.04–0.12	**<0.001**	0.07 (0.02)	0.03–0.11	**0.001**
% FM	0.03 (0.01)	0.01–0.06	**0.004**	0.03 (0.01)	0.00–0.05	**0.029**
FM/FFM	1.33 (0.46)	0.42–2.25	**0.004**	1.04 (0.48)	0.11–1.98	**0.029**
	Total lipids (g/L)			Total lipid intake, (g/24 h)		
BMI (kg/m^2^)	0.01 (0.01)	−0.00–0.02	0.228	−0.00 (0.01)	−0.01–0.01	0.984
FFM (kg)	0.01 (0.00)	0.00–0.01	**0.036**	0.01 (0.01)	−0.00–0.02	0.302
FFMI (kg/m^2^)	0.02 (0.01)	0.00–0.04	**0.030**	0.02 (0.02)	−0.01–0.05	0.312
FM (kg)	0.00 (0.00)	−0.01–0.01	0.789	−0.00 (0.00)	−0.01–0.00	0.385
FMI (kg/m^2^)	0.00 (0.01)	−0.01–0.02	0.868	−0.01 (0.01)	−0.04–0.01	0.342
% FM	−0.00 (0.00)	−0.01–0.01	0.399	−0.01 (0.01)	−0.03–0.00	0.067
FM/FFM	−0.19 (0.18)	−0.53–0.16	0.295	−0.50 (0.25)	−1.00–−0.00	0.050

^1^ Parameter estimate ± standard error (SE): effects of predictors taken from linear mixed models that accounted for postpartum time and time of sample collection. ^2^ Predictors raw *p*-value from linear mixed models. BMI, body mass index; FFM, fat-free mass; FFMI, fat = free mass index; FM, fat mass; %FM, percentage fat mass; FM/FFM, fat mass to fat-free mass ratio; FMI, fat mass index. Bold font indicates significant association.
