# Supplementary material for: Investigation of Relationships between Intakes of Human Milk Total Lipids and Metabolic Hormones and Infant Sex and Body Composition
Source: Nutrients. 2024 Aug 16;16(16):2739. doi: 10.3390/nu16162739 (PMC11357482; doi:10.3390/nu16162739)
Supplement: Supplementary file 1 [file nutrients-16-02739-s001.zip › nutrients-3147353-supplementary.pdf]

Supplementary Materials: Investigation of Relationships between Intakes of the Human Milk Total Lipids and Metabolic Hormones and Infant Sex and Body composition

Majed A. Suwaydi, Ching Tat Lai, Sharon L. Perrella, Jacki L. McEachran, Mary E. Wlodek, Donna T. Geddes and Zoya Gridneva

Supplementary Table S1. Biochemical analyses of human milk components.

Human milk component	Method and equipment	Recovery	Reference
Leptin	Leptin concentration in whole HM samples was analysed using the R&D Systems Human Leptin ELISA DuoSet (DY398, Lot: P262874, R & D system, Minneapolis, MN, USA). The kit was optimized to measure HM leptin using Greiner Bio-One 96-well half area, high binding ELISA plates (Item No.: 675061, Greiner Bio-One GmbH, Austria). Plates were prepared by pipetting 50 µL of capture antibody (4 µg/mL, diluted with PBS, pH 7.4) per well to coat the bottom of the wells of the flat bottom of 96-well ELISA plates and incubated overnight at RT. Plates were washed 3 times with washing buffer (0.05% Tween 20 in PBS, pH 7.4), dispensed at 200 µL per well, using a plate washer (Inteliwasher 3D-IW8 microplate washer, BioSan SIA, Latvia), then blocked by adding 150 µL of reagent diluent (1% BSA in PBS, pH 7.4) and incubated for a minimum 1 h. Next, plates were washed 3 times, and 50 µL of standards (concentration range 0.9 – 0.014 ng/mL), samples and internal quality control were added in duplicate and incubated for minimum 2 h at RT. Then, plates were washed three times and 50 µL of detection antibody (25 ng/mL,	Recovery average of leptin was 97.9 ± 6% (*n* = 5, coefficient of variation (CV) = 2.8%) with a detection limit of 0.014 ng/mL, and the average intra- and inter-assays CV were 1.62% and 10.01%, respectively.	Suwaydi, M.A.; Lai, C.T.; Rea, A.; Gridneva, Z.; Perrella, S.L.; Wlodek, M.E.; Geddes, D.T. Circadian variation in human milk hormones and macronutrients. *Nutrients* **2023**, *15*, 3729, doi:10.3390/nu15173729.

	diluted in reagent diluent, pH 7.4) was added and incubated for minimum 2 h at RT. Following the second incubation, 50 µL of Streptavidin-HRP (Lot: P255687, 40-fold dilution in reagent diluent, pH 7.4) was added and incubated for a minimum of 20 min at RT in a dark area to avoid exposure to direct light. Next, 50 µL of substrate colour reagent, 1:1 mixture of Color Reagent A (H_2O_2) and Color Reagent B (Tetramethylbenzidine) (DY999, R&D Systems, Minneapolis, MN, USA) was added and incubated for 20 min at RT in a dark area to avoid exposure to direct light. The reaction was stopped using 25 µL sulphuric acid stop solution (2 N H_2SO_4, Merck, USA). Absorbance was read at 450 nm by a plate spectrophotometer (Enspire Multimode Plate Reader, Waltham, MA, USA).		
Adiponectin	Adiponectin concentration in whole HM samples was analysed using the Human Adiponectin ELISA, High Sensitivity (RD191023100, Lot: E21-040, BioVendor, Brno, Czech Republic) following the protocol (b) that manufacturer recommended for use to measure HM adiponectin concentration.	Recovery average of adiponectin was 96.2 ± 3.2% (*n* = 10) with a detection limit of 0.156 ng/mL [Gridneva et al., 2018]. The average intra- and inter-assay CV were 6% and 11.97%, respectively.	Suwaydi, M.A.; Lai, C.T.; Rea, A.; Gridneva, Z.; Perrella, S.L.; Wlodek, M.E.; Geddes, D.T. Circadian variation in human milk hormones and macronutrients. *Nutrients* **2023**, *15*, 3729, doi:10.3390/nu15173729. Gridneva, Z.; Kugananthan, S.; Rea, A.; Lai, C.T.; Ward, L.C.; Murray, K.; Hartmann, P.E.; Geddes, D.T. Human Milk Adiponectin and Leptin and Infant Body Composition over the First 12 Months of Lactation. *Nutrients* **2018**, *10*, 1125, doi:10.3390/nu10081125.
Insulin	Insulin concentration in whole HM samples was analysed using the human Insulin ELISA BioVendor (RIS006R, Lot: X21-136S01, BioVendor, Brno, Czech Republic). Following the manufacturer's protocol, 50 µL homogenized milk	Recovery average of insulin was 100.33 ± 2.82% (*n* = 5, CV = 2.81%) with a detection limit	Suwaydi, M.A.; Lai, C.T.; Rea, A.; Gridneva, Z.; Perrella, S.L.; Wlodek, M.E.; Geddes, D.T. Circadian variation in human milk hormones and macronutrients. *Nutrients*

	sample (undiluted), calibrators, controls were pipetted into appropriate wells. Next, 50 µL of anti-insulin HRP conjugate were added and incubated for 30 min. Plates were washed 3 times with washing buffer (Tris-HCl), dispensed at 400 µL per well, using a plate washer (Inteliwasher 3D-IW8 microplate washer, BioSan SIA, Latvia), then 100 µL of chromogenic solution (Tetra-methylbenzidine) was added per well and incubated for minimum 15 min. The reaction was stopped using 100 µL of stopping reagent (2 N HCl).	of 0.17 µIU/mL. The average intra- and inter-assay CV were 2.34% and 15.8%, respectively.	**2023**, *15*, 3729, doi:10.3390/nu15173729.
Total lipids	Fat concentration (%) was measured using the creamatocrit method [Meier et al., 2006], and the cream percentage was converted to g/L using the following formula: **Fat (g/L) = 3.968 + (5.917 × Creamatocrit (%))**	NA	Suwaydi, M.A.; Lai, C.T.; Rea, A.; Gridneva, Z.; Perrella, S.L.; Wlodek, M.E.; Geddes, D.T. Circadian variation in human milk hormones and macronutrients. *Nutrients* **2023**, *15*, 3729, doi:10.3390/nu15173729. Meier, P.P.; Engstrom, J.L.; Zuleger, J.L.; Motykowski, J.E.; Vasan, U.; Meier, W.A.; Hartmann, P.E.; Williams, T.M. Accuracy of a user-friendly centrifuge for measuring creamatocrits on mothers' milk in the clinical setting. *Breastfeed Med* **2006**, *1*, 79-87, doi:10.1089/bfm.2006.1.79.

Abbreviations: ELISA, enzyme-linked immunosorbent assay; NA, not applicable; RT, room temperature.
